# Prevalence of reverse transcriptase and protease mutations associated with antiretroviral drug resistance among drug-naïve HIV-1 infected pregnant women in Kagera and Kilimanjaro regions, Tanzania

**DOI:** 10.1186/1742-6405-5-13

**Published:** 2008-06-21

**Authors:** Balthazar M Nyombi, Carol Holm-Hansen, Knut I Kristiansen, Gunnar Bjune, Fredrik Müller

**Affiliations:** 1 Research Laboratory, Kilimanjaro Christian Medical College, Moshi, Tanzania; 2 International Community Health, Institute of General Practice and Community Medicine, Faculty of Medicine, University of Oslo, Oslo, Norway; 3 Institute of Microbiology, Faculty of Medicine, University of Oslo and Rikshospitalet University Hospital, Oslo, Norway; 4 Division of Infectious Disease Control, Norwegian Institute of Public Health, Oslo, Norway

## Abstract

**Background:**

Access to antiretroviral drugs for HIV-1 infection has increased in sub-Saharan Africa (SSA) during the past few years. Mutations in the HIV-1 genome are often associated with treatment failure as indicated by viral replication and elevated levels of virus in the blood. Mutations conferring resistance to antiretroviral drugs are based on comparing gene sequences with corresponding consensus sequences of HIV-1 subtype B that represents only 10% of the AIDS pandemic. The HIV pandemic in SSA is characterized by high viral genetic diversity. Before antiretroviral drugs become more widely available, it is important to characterize baseline naturally occurring genetic mutations and polymorphisms associated with antiretroviral drug resistance among circulating HIV-1 subtypes.

**Methods:**

The prevalence of mutations associated with antiretroviral drug resistance in protease (PR) and reverse transcriptase (RT) regions among antiretroviral treatment-naïve HIV-1 infected pregnant women was investigated in Bukoba (Kagera) and Moshi (Kilimanjaro) municipalities, Tanzania, between September and December 2005. The HIV-1 *pol *gene was amplified using primers recognizing conserved viral sequences and sequenced employing BigDye chemistry from 100 HIV-1 seropositive treatment-naïve pregnant women and 61 HIV-1 seropositive women who had received a single dose of Nevirapine (sdNVP). Positions 1–350 of the RT and 1–99 of the PR genes were analyzed for mutations based on the Stanford University HIV Drug Resistance Database.

**Results:**

HIV-1 subtypes A, C, D, CRF10_CD and Unique Recombinant Forms (URF) were detected. Primary mutations associated with NRTI and NNRTI resistance were detected among 3% and 4% of treatment-naïve strains, respectively. Primary mutations associated with NRTI and NNRTI resistance were detected in 1.6% and 11.5% of women who had received sdNVP, respectively. None of the primary mutations associated with PI resistance was found. Polymorphisms detected in RT and PR sequences were mainly mutations that are found in the consensus sequences of non-B subtypes

**Conclusion:**

Based on the WHO HIV Drug Resistance Research Network Threshold of less than 5%, the baseline prevalence of primary mutations among treatment-naïve HIV-1 infected pregnant women in Kagera and Kilimanjaro regions was low. The significance of HIV-1 subtype B polymorphic positions with respect to antiretroviral resistance identified among the prevalent HIV-1 subtypes is unknown. More studies addressing the correlation between polymorphic mutations, antiretroviral resistance and clinical outcome are warranted in regions where non-B subtypes are prevalent.

## Background

Tanzania is one of the countries in sub-Saharan Africa (SSA) most affected by the HIV pandemic. It was estimated that by the end of 2005 approximately 6.5% of adults throughout the country were living with the virus. The epidemic is characterized by large differences in the prevalence and incidence of HIV-1 infections between and within the regions [1-3]. Kilimanjaro and Kagera have been among the five regions with the highest prevalence of HIV-1 infections in Tanzania [4]. Heterosexual transmission accounts for majority of HIV-1 infections and 55% of the reported cases in Tanzania are among women [4]. Sentinel centres for HIV-1 surveillance were established at antenatal clinics (ANC) based on the assumption that pregnant women attending the clinics constitute an easily accessible population that represent the sexually active population at large. A study conducted in Kagera showed that surveillance of antenatal care pregnant women as a sentinel population represented the prevalence the HIV-1 infections in the general population [5]. A surveillance study performed in 2003/4 indicated that 9.5% and 6.8% of pregnant women attending ANC were HIV-1 seropositive in Bukoba (Kagera) and Moshi (Kilimanjaro), respectively [2].

HIV-1 is classified into three phylogenetic groups, main (M), outlier (O) and non-M, non-O (N). Group M includes 9 subtypes (A-D, F-H, J and K) and 34 Circulating Recombinant Forms (CRF) [6,7] that are characterized by different geographical distributions. The HIV-1 epidemic in Kilimanjaro and Kagera regions is characterized by subtypes A, C and D, CRF10_CD and unique recombinant forms (URF) [8,9]. The great diversity of HIV-1 poses challenges to accurate viral load determination, the development of diagnostic tools and AIDS vaccines, and monitoring antiretroviral drug resistance [10].

In October 2004 Tanzania initiated a program offering antiretroviral treatment at no cost to persons with HIV infection. The treatment includes Triomune, a combination of two nucleoside reverse transcriptase inhibitors (NRTI) Stavudine (d4T) and Lamivudine (3TC), and a non-nucleoside reverse transcriptase inhibitor (NNRTI) Nevirapine (NVP). WHO estimated that 315,000 persons living with HIV-1 in Tanzania were eligible for antiretroviral drugs in 2005 [11]. The national goal was to provide antiretroviral therapy to 44,000 HIV-1 infected individuals by the end of the same year (National Guidelines for Clinical Management of HIV and AIDS). The National AIDS Control Programme (NACP) reported that 19,600 patients were on antiretroviral by the end of 2005. The target was not reached because few centres were selected to provide antiretroviral therapy and an insufficient number of trained health care workers were available to manage the national care and treatment programme. Gaining access to antiretroviral drugs has implications for improving the quality of life for people living with HIV/AIDS, the prevention of vertical transmission as well as the emergence and transmission of antiretroviral resistant virus strains [12]. Incomplete adherence to antiretroviral therapy has been documented to be a primary contributing factor for treatment failure and the development of antiretroviral resistance among patients in resource-limited settings [13].

Amino acid mutations in protease (PR) and reverse transcriptase (RT) associated with resistance to antiretroviral drugs are identified when sequences are compared to the consesus *pol *sequence of wild-type HIV_HXB2_. HIV-1 subtype B represents 10% of all HIV-1 infections globally [14] and is most prevalent in developed countries. Single mutations that confer antiretroviral resistance are known as "primary mutations" while mutations that must appear in combination with other mutations are termed "secondary mutations" [15]. Over 75% of HIV-1 infections worldwide are found in SSA, and the numerous local epidemics in Africa are characterized by diverse circulating non-B genetic subtypes, CRF and URF [16]. Limited studies have been conducted in SSA to identify mutations associated with antiretroviral drug resistance. It is imperative to investigate and describe mutations associated with resistance to antiretroviral drugs in the RT and PR genes of HIV-1 non-B subtypes and CRF among treatment-naïve populations in SSA. Baseline information gained from these studies will be of importance in the development of algorithms for the interpretation of mutations conferring antiretroviral drug resistance in different geographical regions.

The aim of the present study was to report the baseline prevalence of primary and secondary mutations in PR and RT that are associated with antiretroviral drug resistance in treatment-naive pregnant women living in Kagera and Kilimanjaro regions in Tanzania before extensive use of antiretroviral drugs is implemented in the country.

## Methods

### Study subjects and samples

Two hundred forty six HIV-1 infected pregnant women diagnosed seeking antenatal care and women attending postnatal care in Bukoba (Kagera) and Moshi (Kilimanjaro) municipalities were included in the study. All women underwent pre- and post-test counselling and signed informed consent form prior to participating in the study. Inclusion criteria were: (i) HIV-1 seropositive pregnant woman, (ii) pregnant women attending antenatal clinic (ANC), counselled and enrolled in prevention of mother-to-child HIV-1 transmission (PMTCT) program, (iii) not receiving highly active antiretroviral therapy (HAART), (iv) HIV-1 seropositive women attending postnatal care, enrolled in PMTCT and received sdNVP, and (v) signed informed consent form. Blood samples and demographic information were collected during September to December 2005. Plasma was aliquoted and stored at -80°C immediately after separation.

HIV-1 serology was performed based on the national HIV testing algorithm using the two rapid assay confirmatory strategy for the detection of antibodies to HIV [17]. HIV antibody testing was performed at the clinics by counsellors using two rapid assays, Capillus (Trinity Biotech, Ireland) and Determine (Abbott Laboratories, IL, USA). Discordant blood samples were sent to the main laboratories in Bukoba (Kagera) or Mawenzi and KCMC (Kilimanjaro) for additional testing using ELISA (Vironostika^® ^HIV Uni-Form II Ag/Ab ELISA test; bioMérieux, France). Plasma samples were shipped on dry ice to the Institute of Microbiology at Rikshospitalet in Oslo, Norway for further laboratory analysis.

The study was reviewed and approved by the KCMC Ethics Committee (Tanzania) and the Regional Committee for Ethics in Medical Research (Norway) in compliance with institutional policies and national guidelines.

### Viral load, RNA extraction, RT PCR, secondary PCR and sequencing

Viral load was determined in 1 ml of plasma using COBAS AmpliPrep (ROCHE Molecular Systems, Inc. Branchburg, NJ, USA). HIV-1 RNA was extracted from 1 ml of plasma using COBAS AmpliPrep Total Nucleic Acid Isolation kit (ROCHE) or by the method developed by Boom R. et al. [18] for plasma samples that had volumes less than 1 ml or viral load below 10,000 particles/ml. RT PCR for the *pol *region was performed using the Qiagen One-Step RT PCR kit (QIAGEN, Valencia, CA, USA) with primer pair P2073G/P3709. Secondary PCR was performed using primer pair P2073/P3681 under conditions recommended by the manufacturer. Detailed RT PCR and secondary PCR parameters are described elsewhere [19]. Ultrapure Water was used as a negative control in each run.

PCR products were purified using MicroSpin columns (GE Healthcare BioSciences, Piscataway, NJ, USA). Amplicons were sequenced employing BigDye chemistry sequence kit (Applied Biosystems, Foster City, CA, USA.) on an ABI Prism 3730 DNA sequencer (Applied Biosystems) using primers P3, P10, P11, P12, P13, P14, P15 and P3681 according to the manufacturer's protocol as previously described [19].

Generated sequences were assembled using Sequencher version 4.5 software (Gene Codes Corporation) and the resulting contig sequences were edited manually. The sequences were analyzed by bootscan and similarity analysis using SimPlot version 3.5.1 [20]. Multiple alignments of the edited sequences with reference sequences from the HIV sequence database of the Los Alamos National Laboratory were performed using ClustalX [21]. Kimura two-parameter analysis was applied to calculate the evolutionary distance, and phylogenetic trees were constructed using the neighbour-joining method. Bootstrap values of 100 were used and sequences were gap stripped prior to tree construction (data not shown). Phylogenetic trees were viewed using TreeView (Win32) version 1.6.6 (Institute of Biomedical and Life Science, University of Glasgow, UK).

### Drug resistance analysis

The edited sequences were submitted to Stanford University HIV Drug Resistance Database and drug resistance mutation profiles were analysed based on HIVdb: Genotypic Resistance Interpretation Algorithm [22].

### Statistical methods

A statistical comparison of different group proportions was made using the Chi square test and Fisher's exact test when appropriate. Level of significance was set to p ≤ 0.05.

## Results

### Study samples

A total of 161 HIV-1 *pol *gene sequences, 64 from Bukoba (Kagera) and 97 from Moshi (Kilimanjaro) were successfully generated. One hundred were from antiretroviral treatment-naïve women (36 from Kagera and 64 from Kilimanjaro) while 61 women (28 from Kagera and 33 from Kilimanjaro) received sdNVP during their previous deliveries. Of the 85 samples that were not successfully sequenced 16 had no detectable viral particles while 69 had a low level of viral particles. The time between sdNVP and sample collection for this study ranged from 1 to 48 months in both regions. Women were attending the postnatal clinic 1 month after delivery while a few pregnant women seeking antenatal care reported that they received sdNVP during their previous pregnancy. Table 1 in Additional file 1 shows the baseline characteristics of study subjects in both regions.

### HIV-1 pol subtyping

**Figure 1:**
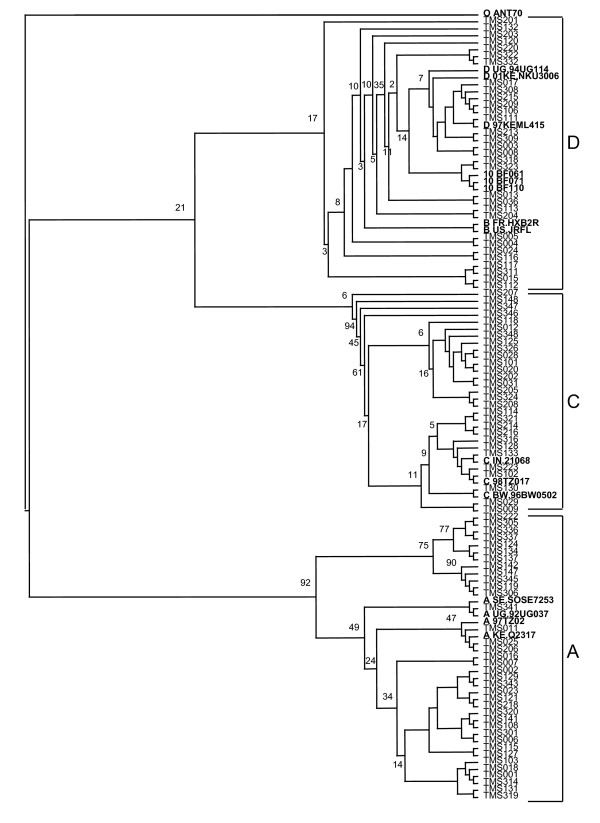
**Phylogenetic analysis of HIV-1 pol sequences from HIV-1 infected pregnant women in Moshi, Kilimanjaro**. The study and reference sequences were aligned by the Cluster X programme and tree constructed by neighbour-joining method based on the Kimura two-parameter and viewed using TreeView software Sequence O ANT70 was used as out-group for phylogenetic analysis.

**Figure 2:**
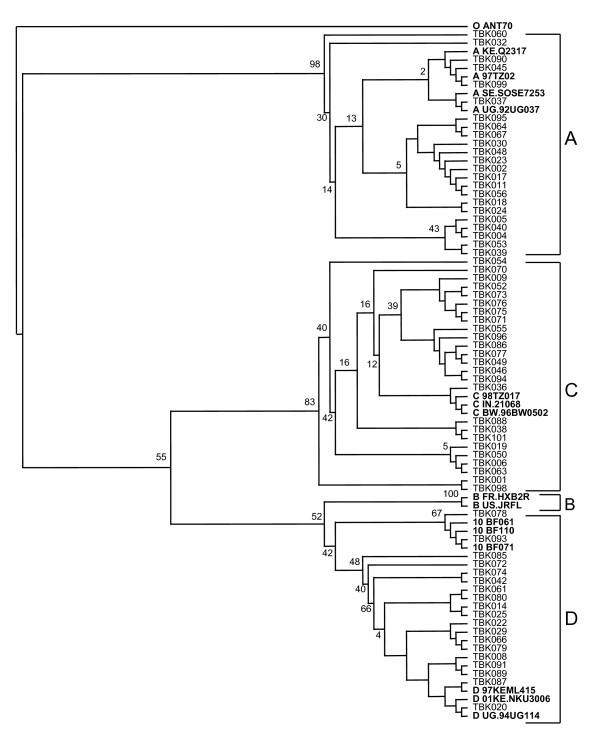
**Phylogenetic analysis of HIV-1 pol sequences from HIV-1 infected pregnant women in Bukoba, Kagera**. The study and reference sequences were aligned by the Cluster X programme and tree constructed by neighbour-joining method based on the Kimura two-parameter and viewed using TreeView software Sequence O ANT70 was used as out-group for phylogenetic analysis.

HIV-1 *pol *subtypes in both regions were A (34%), C (26%), D (19%), URF (19%) and CRF10_CD (2%). URF sequences clustered with various reference sequences representing one of the recombining subtypes in the phylogenetic analysis. The phylogenetic trees are presented in Figures 1 and 2. Distribution of HIV-1 subtypes in the two regions is summarized in Table 1 in additional file 1.

### Protease inhibitor (PI) resistance-associated mutations

None of the known primary mutations associated with PI resistance (D30N, V32I, V33F, M46IL, I47VA, G48V, I50VL, I54VALM, L76V, V82AFTSL, I84V, N88DS, L90M) were detected among the PR sequences from the two regions. Secondary mutations V11I (1), K20R (19), L23I (1), L33F (2), L33I (1), L33IV (1), E35G (1) and polymorphic mutations L10I (8), L10V (6), A71T (3) were observed in 20 (12.4%) of the 161 sequences. Polymorphic mutation at position 63 showed a great variation of amino acid substitutions (L63P/T/S/P/Q/M/A/V/C/H/N/L). Mutations L10I (5), L10V (1), V11I (1), K20R (8), L33F (2), A71T (1) and L10I (4), L10V (5), K20R (11), L23I (1), L33I (1), L33IV (1), E35G (1), A71T (2) were detected among sequences from Kagera and Kilimanjaro strains, respectively. One sequence from Kilimanjaro strain had an atypical PI resistance-associated primary mutation, G48K, but this mutation confers no reduction in susceptibility to PIs.

### Reverse transcriptase inhibitor (RTI) resistance-associated mutations among treatment-naïve HIV-1 infected women

Primary mutations associated with NRTI were found at positions V118I (3%) and T69D (1%) while secondary mutations were observed at positions T69S (4%) and G333E (2%). Primary mutations associated with NNRTI were found at positions E138K (2%), V179E (1%) and P225H (1%) while a secondary mutation was observed at position K103R (1%). Primary mutations associated with NNRTI resistance were not found in subtype A strains while primary mutations associated with NRTI resistance were not found in subtype D strains among treatment-naïve pregnant women included in this study. Thymidine analogue mutations (TAM) were not detected in all strains from both regions. Table 2 in additional file 1 shows the distribution of resistance mutations among HIV-1 *pol *subtype sequences amplified from strains obtained from treatment-naïve pregnant women attending antenatal clinics and those who received sdNVP in Bukoba and Moshi municipalities in Kagera and Kilimanjaro regions, respectively.

### RTI resistance-associated mutations in HIV-1 infected women exposed to sdNVP

Primary mutations associated with NRTI were found at position M184I (1.6%) while secondary mutations were observed at positions G333E (1.6%) among woman who had received sdNVP. Primary mutations associated with NNRTI were found at positions V179D (3.3%), K103N (1.6%), K103T (1.6%), Y181C (1.6%), G190A (1.6%), and K238N (1.6%) while a secondary mutation was observed at position K103R (3.3%).

None of the secondary mutations associated with NNRTI resistance was detected among the strains from Kagera.

### Polymorphisms among treatment-naïve subtype A, C and D strains

Subtype B-associated naturally occurring polymorphisms identified in RT sequences were E36A, S48T, K49R, V60I, F177E, V179I, T200I/L/R/A, Q207A/E/K/D, R211S/K, F214Q/K/T/N/L, L282C, L283I, P294T, E312D/N, P345Q and F346Y. Polymorphic amino acid residuals that are found in consensus sequences of subtype A (V60I, Q207A, R211S, P294T, E312D), subtype C (E36A, S48T, T200A) and subtype D (K49R, V60I) were detected in at least 60% of the RT sequences. Similarly, non-consensus polymorphic amino acid residuals Q207A and Q207K were detected in subtype C sequences while residuals T200I, R211K, L282C, L283I, P345Q and F346Y were detected in subtype D sequences.

Subtype B associated polymorphisms observed in PR were T12S, I13V, K14R, I15V, E35D, M36I, R41K, K45R, R57K, D60E, I64V, H69K, L89M and I93L. Polymorphic mutations that are found in consensus sequences were observed in more than 90% of all subtypes: in subtype A (I13V, E35D, M36I, R41K, H69K and L89M), subtype C (T12S, T15V, M36I, R41K, H69K, L89M and I93L) and subtype D (M361, R41K). Non-consensus polymorphic amino acid residuals I13V and I64V were observed in subtype D while R41N was observed in subtype C.

## Discussion

Data addressing the prevalence of primary mutations associated with antiretroviral resistance among treatment-naïve individuals infected with HIV-1 in SSA is limited. Reports from Uganda [23,24], Rwanda [24], Mozambique [25] and Zambia [26] indicated a low prevalence of primary mutations associated with antiretroviral resistance among treatment-naïve individuals infected with HIV-1 subtypes A, C and D stains. Our study provides the first report of primary mutations associated with antiretroviral resistance among antiretroviral treatment-naïve pregnant women in Tanzania. In accordance with other reports from SSA, a low prevalence of primary mutations associated with antiretroviral resistance was found among treatment-naïve pregnant women living in Kilimanjaro and Kagera regions. We have previously reported the similar prevalence of HIV-1 *pol *subtypes circulating in these 2 regions [19].

Only one primary mutation associated with NRTI resistance, V118I, which causes low-level resistance to Lamivudine (3TC) and possibly other NRTIs when present in combination with E44E/D, was observed in three strains from Kilimanjaro. However, one atypical mutation, V118C, was detected in one strain; the significance of this mutation is not yet known. Primary mutations associated with resistance that cause low-level resistance to NNRTI in three strains (Two E138K and V179E) from Kilimanjaro were detected among treatment-naïve pregnant women. Further, no primary mutations associated with PI resistance were detected in this study. The low prevalence or lack of primary mutations associated with antiretroviral resistance in Kilimanjaro and Kagera, respectively, reflects the limited use of antiretroviral drugs in the two regions to date. The absence of TAM among the strains investigated may indicate the low use of the AZT-based therapy in the population living in these regions. Secondary mutations associated with NRTI resistance at position 69 (2 from Kagera and 4 from Kilimanjaro) and position 333 (2 from Kilimanjaro) were detected. These polymorphisms may confer resistance to Zidovudine (AZT). Secondary mutations K103KR and P225H that are associated with NNRTI resistance were detected in two strains from Kilimanjaro. No secondary mutations associated with NNRTI resistance were detected in Kagera. The majority of the sequences analyzed in this study bore secondary resistance mutations that represent consensus sequences in non-B viruses as was observed in Zambia for subtype C [26]. Polymorphic mutations that are non-consensus in non-B subtype *pol *sequences were detected in both RT and PR. These observations may indicate subtype-specific polymorphic mutations. Further investigations are warranted to elucidate the significance of these mutations with respect to antiretroviral drug resistance. L63P appears to be a general up-regulator of HIV-1 protease activity. The considerable variation in amino acid substitutions (T, S, P, Q, M, A, V, C, H, N and L) that was observed at position 63 in 57% of the sequences raises questions regarding the significance of susceptibility and interpretation of resistance to PIs among non-B strains.

To date most of the reported RT and PR genotypic polymorphisms among non-B strains are based on phenotypic data from antiretroviral-naïve strains [27,28]. *In vitro *antiretroviral susceptibility testing has shown discrepant results as compared to genotypic interpretation of polymorphic mutations in non-B stains [27,29]. Therefore, it is imperative to investigate these polymorphisms in relation to clinical outcome in patients treated with the three-drug combination in Triomune that is the recommended treatment strategy in resource-limited countries such as Tanzania where non-B subtypes are prevalent.

Table 2 in additional file 1 shows the characteristics of strains that showed primary and secondary mutations among treatment-naïve participants and those exposed to sdNVP. The PMTCT programme in Tanzania administers sdNVP to reduce vertical transmission from HIV-1 infected mothers to their offspring. The prevalence of NNRTI primary mutations was higher (p = 0.032, Fisher's exact test) among virus exposed to sdNVP (6/33) compared to treatment-naïve virus (3/64) from Kilimanjaro region. However, no statistical difference was observed when compared to the two groups of strains (1/28 and 0/36) from Kagera region (p = 0.437). There was no statistical difference between the prevalence of NNRTI primary mutations among strains from treatment-naïve pregnant women in Kilimanjaro (3/64) and Kagera (0/36) regions (p = 0.258). Furthermore, there was no statistical difference between the prevalence of NNRTI primary mutations among strains from women received sdNVP in Kilimanjaro (6/33) and Kagera (1/28) regions (p = 0.0768). The differences observed between the two groups of women and the higher prevalence ratio in Kilimanjaro may be explained by the extended and well-established PMTCT centers in Kilimanjaro. Such services are not as developed in Kagera.

Six of seven women with NNRTI primary mutations in both regions received sdNVP between 1 and 8 months before samples were collected for this study. The risk of virological failure in women initiating HAART after sdNVP is associated with the time interval between receiving sdNVP and initiating HAART [30] These results indicate that it is advantageous to provide NVP-containing HAART to HIV-1 infected pregnant women who received sdNVP in Kilimanjaro and Kagera regions.

Information concerning drug status including exposure to sdNVP and/or absence of exposure to any other antiretroviral drugs was self-reported by the study participants. The reliability of self-reported information may be a limitation to our findings due to under- or over-reporting by some of the study participants regarding self-medication.

## Conclusion

The results of this first study reveals the that the baseline prevalence of primary mutations that confer resistance to antiretroviral drugs in treatment-naïve HIV-1 infected pregnant women in Kagera and Kilimanjaro regions is low, perhaps due to a low level of exposure of HIV infected individuals in these regions to antiretroviral drugs. The results of this study need to be confirmed by investigations in different regions of the country. Furthermore, additional studies addressing biological characteristics, the fitness of viral strains carrying polymorphic mutations and the evolution of HIV-1, all issues that may be relevant for public health measures in Tanzania, are warranted.

### Sequence data

Sequences were submitted to GenBank and accession numbers assigned [EU251715 – EU251732; EU251734 – EU251746; EU251748 – EU251754; EU251756 – EU251766; EU251768 – EU251821; EU251823 – EU251846; EU251848 – EU251874; and EU251876 – EU251881].

## Competing interests

The authors declare that they have no competing interests.

## Authors' contributions

BMN, CHH and FM conceived the study, BMN coordinated data collection and performed laboratory, data analyses and interpretation of results, GB participated in study design, KIK coordinated laboratory analysis, and KIK and FM participated in laboratory analysis, FM and CHH participated in study design, interpretation of results. All authors contributed to the writing, read and approved the final manuscript.

## Supplementary Material

Additional file 1 **Table 1.** Characteristics of 161 HIV-1seropositive pregnant women included in a study for the prevalence of primary and secondary mutations associated with antiretroviral drug resistance conducted from September to December 2005 in Kagera and Kilimanjaro regions, Tanzania. Click here for file
