# Supplementary material for: Prevalence of reverse transcriptase and protease mutations associated with antiretroviral drug resistance among drug-naïve HIV-1 infected pregnant women in Kagera and Kilimanjaro regions, Tanzania
Source: AIDS Res Ther. 2008 Jun 21;5:13. doi: 10.1186/1742-6405-5-13 (PMC2443165; doi:10.1186/1742-6405-5-13)
Supplement: Additional file 1 — Table 1. Characteristics of 161 HIV-1seropositive pregnant women included in a study for the prevalence of primary and secondary mutations associated with antiretroviral drug resistance conducted from September to December 2005 in Kagera and Kilimanjaro regions, Tanzania. [file 1742-6405-5-13-S1.doc]

Table 1. Characteristics of 161 HIV-1seropositive pregnant women included in a study for the prevalence of primary and secondary mutations associated with antiretroviral drug resistance conducted from September to December 2005 in Kagera and Kilimanjaro regions, Tanzania.

Characteristic Kagera Kilimanjaro TOTAL

Treatment-naïve sdNVP TOTAL Treatment-naïve sdNVP TOTAL

(n=36) (n=28) (n=64) (n=64) (n=33) (n=97) n=161

CD+4 -Median 427 463 434 356 292 331

(cells/cmm)

-Range 142 -959 77 -1195 77 - 1195 49 -1092 15 – 714 15 - 1092

Viral load -Median 68000 28200 38750 19500 30000 19600

(copies/ml)

-Range (2430 – 4360000) (298 – 411000)(298 – 4360000) (191 -3875000) (40 – 2670000) (40 – 3875000)

Age -Median 25 31 28 27 30 28

(yrs)

-Range 15-45 20-41 15-45 17-47 19-41 17-47

Subtypes A 20 (31%) 35 (36%) 55 (34%)

[n (%)] C 17 (27%) 24 (25%) 41 (26%)

D 12 (19%) 19 (19%) 31 (19%)

CRF10_CD 2 (3%) 1 (1%) 3 (2%)

URF 13 (20%) 18 (19%) 31 (19%)

sdNVP: single dose Nevirapine; URF: Unique recombinant forms; n (%): number (percentage)

Table 2. Mutations associated with NRTI and NNRTI resistance among treatment-naïve pregnant women and those received sdNVP attending antenatal in Kagera and Kilimanjaro regions.

Sample ID Age Viral load Time post Subtype NRTI NNRTI Type of

(years) (copies/mL) sdNVP mutation

TBK023 23 23000 AN A T69S - S

TBK076 35 16600 AN C T69S - S

TMS343 39 652 AN A T69S - S

TMS216 29 12300 AN C V118C* - S

TMS348 26 16200 AN C G333E - S

TMS119 30 3875000 AN AD T69S - S

TMS120 35 452500 AN D G333E - S

TMS002 23 2113 AN A V118I - P

TMS114 27 773 AN C V118I - P

TMS321 22 4500 AN C V118I, T69D - P, P

TMS028 25 452 AN C - K103R S

TMS326 35 14600 AN C - P225H P

TMS205 31 2300 AN C - V179E P

TMS003 27 26400 AN D - E138K P

TMS008 28 16900 AN D - E138K P

TBK019 28 2520 8 months C - K103N P

TMS013 20 9160 15 months CRF10_CD G333E - S

TMS206 21 2310 48 months A - K238N P

TMS023 34 40 8 months A M184I G190R* P, S

TMS208 26 366000 7 months C - K103R S

TMS323 32 674 2 months D - V179D P

TMS332 30 2520000 1 month D - K103T P

TMS111 27 1620 1 month D - K103R, V179D** S, P

TMS311 25 2670000 1 month AD - Y181C, G190A P, P

Abbreviations: AN = antiretroviral drug-naïve, P = primary mutation; S = secondary mutation; TBK = sample from Kagera;

TMS = sample from Kilimanjaro; NRTI = nucleoside/nucleotide reverse transcriptase inhibitor; NNRTI = non-nucleoside reverse transcriptase inhibitor

* Atypical mutation at position 118 and 190

** The combination of K103R + V179D reduces susceptibility of NNRTI
